# Association between serum angiotensin-converting enzyme 2 levels and postoperative myocardial infarction following coronary artery bypass grafting

**DOI:** 10.3892/etm.2014.1640

**Published:** 2014-03-27

**Authors:** MIN WANG, WEI ZHANG, YU ZHOU, XINMIN ZHOU

**Affiliations:** 1 Department of Cardiothoracic Surgery, The Second Xiangya Hospital, Central South University, Changsha, Hunan 410011, P.R. China; 2 The Second Xiangya Hospital, Central South University, Changsha, Hunan 410011, P.R. China

**Keywords:** angiotensin-converting enzyme 2, coronary artery bypass grafting, myocardial infarction, cardiopulmonary bypass, hazard ratio, gene polymorphism

## Abstract

Myocardial infarction (MI) is a predominant and severe complication in patients that undergo aortocoronary bypass surgery. Angiotensin-converting enzyme 2 (ACE2) activation is reportedly a protective mechanism in MI; therefore, in the present study, the association between serum ACE2 levels and postoperative MI following coronary artery bypass grafting (CABG) was investigated. Preoperative and postoperative serum ACE2 levels in 136 subjects undergoing CABG were observed and the serum ACE2 levels, 1 h post surgery, were divided into quartile categories. Following adjustment for age, gender, body mass index, hypertension, previous MI, current smoking status, hyperlipidemia, diabetes mellitus, Gensini score, aortic clamp time, number of grafts and pre-CABG medications; the risk of developing postoperative MI following CABG was observed to be significantly higher in the lowest serum ACE2 level quartile than when compared with the highest quartile (hazard ratio, 2.94; 95% confidence interval, 1.85–4.16; P=0.009). The subjects that exhibited a serum ACE2 level ≤1.06 ng/ml showed significantly higher rates of postoperative MI, arrhythmia and reduced cardiac output in addition to increased instances of in-hospital mortality post CABG, compared with those exhibiting a serum ACE2 level >1.06 ng/ml. A significant negative correlation was observed between serum ACE2 and serum cardiac troponin I levels, however, no significant association was identified between the serum ACE2 level quartiles and the ACE2 gene polymorphisms. The present study indicated that a low serum ACE2 level, 1 h post CABG was independently associated with an increased risk of postoperative MI. Thus, the serum ACE2 level may be a potential novel prognostic factor for postoperative MI following CABG.

## Introduction

Despite substantial advances in surgical, cardioprotective and anesthetic techniques, the incidence of myocardial infarction (MI) following cardiac surgery remains between 3 and 15% and is associated with reduced, long-term survival rates (1). Postoperative MI is a predominant and severe complication in patients undergoing aortocoronary bypass surgery and is a multifactorial disorder with significant inter-patient variability, which is poorly predicted by clinical procedures (1). The development of MI following cardiac surgery has been associated with high morbidity, mortality and cost (2).

The renin-angiotensin system (RAS) is crucial in cardiovascular regulation (3). In the RAS, angiotensin-converting enzyme (ACE) metabolizes angiotensin (Ang) I to form Ang II, which exerts a direct trophic effect upon cardiovascular cells (3). Local Ang II production is important in the pathophysiology of the RAS in the cardiovascular system (4) and recently, ACE2, a novel member of the RAS, was identified to function as a negative regulator of the Ang system by metabolizing Ang II into a putatively protective peptide Ang (1–7) exhibiting high efficiency (5–7). ACE2 is present in the heart and a reduction in its expression is associated with enhanced cardiac hypertrophy and reduced pumping ability (8,9). Although ACE2 was initially localized exclusively in the cardiac endothelial cells, more recent studies have demonstrated ACE2 immunoreactivity in the endothelial and smooth muscle cells of the myocardial vessels, as well as within cardiomyocytes (8,9). Following MI, significant activation of cardiac ACE2 occurs in rats and humans, which combats the adverse effect of an activated cardiac RAS (8). Further evidence of the cardioprotective role of ACE2 arises from studies conducted on ACE2-knockout mice, where the loss of ACE2 facilitated adverse, post-MI ventricular remodeling (10); furthermore, previous studies have indicated that ACE2 overexpression in MI rats improved cardiac contractility and remodeling (10,11). It has been identified that ACE2 serum activity increases during the first week following an acute MI and that ACE2 activation may be a compensatory mechanism in MI (12).

In the present study, the association between serum ACE2 levels and postoperative MI, following coronary artery bypass grafting (CABG), was investigated.

## Patients and methods

### Patients

Between March 2008 and April 2013, 136 Han Chinese patients, who underwent CABG with a cardiopulmonary bypass (CPB) at the Department of Cardiothoracic Surgery of the Second Xiangya Hospital, Central South University (Hunan, China) were enrolled in the present study. The exclusion criteria were a history of renal failure, active liver disease, bleeding disorders, autoimmune diseases or immunosuppressive therapy; patients with a family history of coronary artery disease were also excluded. The present study was approved by the Ethics Committee of the Second Xiangya Hospital, Central South University and the participants provided written informed consent prior to commencing the study.

### Definition of postoperative MI

According to definitions for periprocedural necrosis and periprocedural infarction, collectively established by the American College of Cardiology Foundation, American Heart Association, European Society of Cardiology and the World Heart Federation taskforce, cardiac troponin I (cTnI) is the preferred biomarker for MI. Post-CABG biomarker values that exceed the 99^th^ percentile of the normal reference range represent myocardial necrosis (13) and in the present study, a postoperative MI was defined as an increase of cTnI to greater than five times the 99^th^ percentile of the normal reference range, during the first 72 h following CABG. This was in addition to manifestations of novel pathological Q-waves, a left bundle branch block, an angiographically documented novel graft of native coronary artery occlusion or imaging evidence of a novel loss of a viable MI (13).

### Data collection

Patients that were undergoing CABG ceased all antiplatelet agent usage at least two days prior to surgery. Intraoperative anesthetic, perfusion and cardioprotective management was standardized, using fentanyl-isoflurane anesthesia, nonpulsatile CPB (32–35°C), crystalloid prime, pump flow rates >2.4 l/min/m^2^, cold blood cardioplegia, α-stat blood gas management, heparin (to maintain activated clotting times >450 secs), ɛ-aminocaproic acid infusion and serial hematocrit levels were maintained at ≥0.18 during the CPB. The serum ACE2 levels were measured using an ACE2 (human) ELISA kit (K4918-100; BioVision, Milpitas, CA, USA) according to the manufacturer’s instructions on preoperative day 3 and 1, 1 h post surgery and postoperative days 1, 2, 6, 9 and 12. Serum creatine kinase (CK)-MB levels were measured via a human cTnI ELISA kit (EA-0301; Signosis, Sunnyvale, CA, USA) according the manufacturer’s instructions on preoperative day 1, 1 h post surgery and postoperative days 1, 2, 6 and 9.

### Genotyping

Three single nucleotide polymorphisms (SNPs), 1075A/G (rs1978124), 8790A/G (rs2285666) and 16854G/C (rs4646142) were selected as proxies to investigate the ACE2 polymorphisms as previously described (14). Quality control was performed by sequencing the three SNPs in 80 randomly selected subjects from the study cohort; the discrepancy rate was 1.25%.

### Statistical analysis

Serum ACE2 levels were divided into quartile categories; ≤1.06, 1.07–1.42, 1.43–1.85 and ≥1.86 ng/ml. The adjusted hazard ratios (HRs) and the 95% confidence intervals (CIs) were calculated using the Cox proportional hazard model. The continuous variable values were expressed as the mean ± standard deviation and comparisons between the means of the two groups were performed using Student’s t-test. The categorical variables were expressed as n (%) and analyzed using the χ^2^ test. Statistical analysis was performed using SPSS version 10.0 (SPSS Inc., Chicago, IL, USA). The statistical significance level of this study was set at a two-tailed α=0.05.

## Results

### Serum ACE2

**Figure 1:**
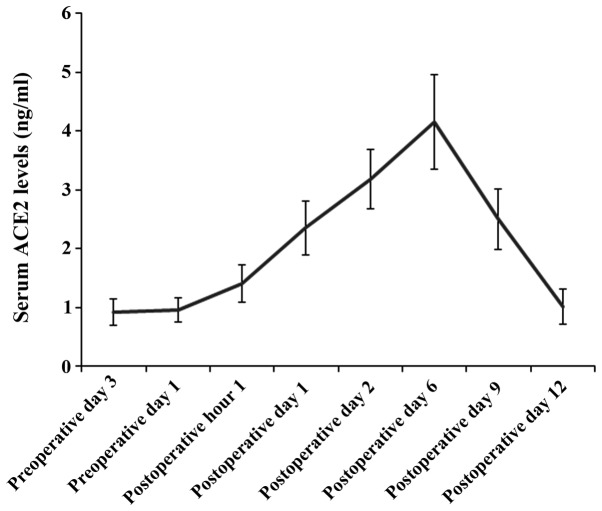
Preoperative and postoperative serum ACE2 levels in patients undergoing coronary artery bypass grafting. ACE2, angiotensin-converting enzyme.

The serum ACE2 levels were measured in the blood samples collected on preoperative days 3 and 1, 1 h post surgery, and postoperative days 1, 2, 6, 9 and 12. The serum ACE2 level observed in the patients who underwent CABG with CPB, was at baseline level prior to surgery and began to rise 1 h post CABG; six days after surgery, the serum ACE2 levels peaked and subsequently returned to baseline 12 days post surgery (Fig. 1). To investigate the prognostic value of the serum ACE2 level for postoperative MI following CABG, the serum ACE2 level was analyzed 1 h post surgery; this was the earliest time point at which the serum ACE2 level began to rise above baseline following CABG. The serum ACE2 levels that were observed 1 h post surgery, were divided into quartile categories; ≤1.06, 1.07–1.42, 1.43–1.85 and ≥1.86 ng/ml. No statistically significant differences were identified between the quartile categories in age, gender, body mass index (BMI), current smoking status, unstable angina, prior MI and prevalence of hyperlipidemia, hypertension and diabetes mellitus (Table I). Furthermore, there were no statistically significant differences observed between the quartile categories regarding disease pattern, procedural characteristics and pre-CABG medication use (Table II).

### Postoperative MI increases following a decrease in the levels of serum ACE2

Postoperative MI increased as the serum ACE2 level decreased and the risk was identified to be significantly higher in the first quartile group when compared with the second, third and fourth quartile groups (Model 1; Table III). Following adjustment for age, gender, BMI, hypertension, prior MI, current smoking status, hyperlipidemia, diabetes mellitus, Gensini score, aortic clamp time, number of grafts and pre-CABG medications, the risk of developing postoperative MI following CABG was significantly higher in the lowest serum ACE2 level quartile group, compared with the highest quartile group (hazard ratio, 2.94; 95% CI, 1.85–4.16; P=0.009). Analysis of serum ACE2 levels on postoperative days 1, 2, 6 and 9 resulted in values that were not statistically significant (data not shown). All of the subjects exhibited significant negative correlation between serum ACE2 and cTnI levels 1 h after surgery, and on days 1, 2, 6 and 9 post surgery, ranging between r=−0.525 (1 h prior to surgery; P<0.001) and r=−462 (postoperative day 9; P<0.001).

Association between low serum ACE2 levels and typical morbidities postCABG. The association between the lowest serum ACE2 level quartile group (≤1.06 ng/ml) and the incidence of typical morbidities post CABG, including postoperative MI, arrhythmia, low cardiac output, pulmonary complications, neurological complications and excessive bleeding was investigated. In the analysis, the second, third and fourth quartiles of serum ACE2 levels were combined into a single category (>1.06 ng/ml; n=102) to enable comparison with the first serum ACE2 level quartile group (≤1.06 ng/ml; n=34). The subjects exhibiting a serum ACE2 level ≤1.06 ng/ml indicated significantly higher rates of postoperative MI, arrhythmia and reduced cardiac output, as well as greater in-hospital mortality following CABG, compared with those exhibiting a serum ACE2 level >1.06 ng/ml (Table IV).

### ACE2 polymorphisms

ACE2 polymorphisms are reportedly associated with MI; Yang *et al* (14) reported that ACE2 SNPs, 1075A/G (rs1978124), 8790A/G (rs2285666) and 16854G/C (rs4646142) were associated with MI (14). Association analysis that was conducted with female subjects indicated that the 1075AA and 16854GG genotypes were significantly associated with MI (P<0.05) and that the 8790AA genotype was associated with MI at a non-significant level (P=0.058). In the male subjects, the 1075A/G-8790A/G and 16854G/C haplotype GGC was significantly associated with MI, when compared with the most common haplotype AAG (P<0.05) (14). To determine whether those ACE2 polymorphisms were associated with the serum ACE2 level in the present study, the association between the serum ACE2 level and the ACE2 genotypes and haplotypes, reportedly associated with MI, were examined in all of the subjects. As the ACE2 gene is located on the X chromosome, the association analysis was conducted by gender. There was no significant association observed between the serum ACE2 level quartile groups and the ACE2 1075AA, 16854GG or 8790AA genotypes in the female subjects, or the ACE2 1075A/G-8790A/G and 16854G/C haplotype GGC in the male subjects (Table V). The results indicated that the observed association between the serum ACE2 level and postoperative MI following CABG, in the present study, was not a result of ACE2 gene polymorphisms.

## Discussion

MI is a predominant and severe complication in patients undergoing aortocoronary bypass surgery. Early diagnosis and prediction of MI is an important condition for optimal postoperative patient management. Previous studies have indicated that ACE2 activation is a protective mechanism in MI and that supplementing ACE2 may be a potential therapy for MI/ischemic heart disease (8–11,15). In the present study, the results indicated that the serum ACE2 level was associated with postoperative MI and in-hospital mortality following CABG.

The serum ACE2 level remained at baseline level preoperatively, began to rise 1 h post CABG, peaked on day 6 post surgery and returned to baseline level on day 12, post surgery. As MI can occur within 24 h of CABG, the serum ACE2 level 1 h post surgery was analyzed to evaluate the potential prognostic value of the serum ACE2 level for postoperative MI following CABG. The association analysis results 1 h post surgery were significant compared with those at other time points. According to previous studies, the duration of aortic cross-clamping, number of coronary grafts and history of previous cardiac surgery were independent predictors of postoperative MI (1). In addition, ACE inhibitors and angiotensin receptor blockers are capable of enhancing ACE2 expression (16,17). Thus, the hazard ratio was adjusted for prior MI, aortic clamp time, number of grafts and pre-CABG medications, in addition to a variety of other confounding factors that may have affected the incidence of postoperative MI and/or serum ACE2 levels, including age, gender, BMI, hypertension, current smoking status, hyperlipidemia, diabetes mellitus and Gensini score. After adjusting for the confounding factors, the risk of developing postoperative MI remained significantly higher in the lowest serum ACE2 level quartile group than in the highest quartile group, 1 h post surgery, indicating that the serum ACE2 level 1 h post surgery may be an independent risk factor for postoperative MI following CABG. Thus, the serum ACE2 level 1 h post surgery may be a potential novel biomarker or prognostic factor for postoperative MI following CABG; its value in clinical applications may be investigated in future studies using a larger patient population.

Postoperative MI, following cardiac surgery is associated with reduced long-term survival rates (1). In the present study, the serum ACE2 level in the lowest quartile group was observed to be associated with the increased rate of postoperative MI, arrhythmia, low cardiac output and in-hospital mortality, indicating that the serum ACE2 level may be a potential novel prognostic factor for short-term survival following cardiac surgery. A prospective study, using the same patient cohort, is being conducted to investigate the association between the serum ACE2 level, postoperative MI and long-term survival rate following CABG.

cTnI, a contractile protein unique to the heart muscle, is a sensitive biomarker, which was introduced predominantly for risk stratification in patients exhibiting acute coronary syndrome and is the gold standard for identifying MI (18,19); in addition, ACE2 is expressed in the heart and has been observed to exhibit a protective effect during MI (8–11). Increasing evidence indicates that the expression of cardiac ACE2 is higher following MI, which combats the adverse effects of an activated cardiac RAS and, therefore, may be a compensatory mechanism in MI (8–11,15,20). In agreement with this, a significant increase in the ACE2 serum level following MI was observed in the present study. Notably, postoperative serum ACE2 and cTnI levels exhibited significant negative correlation, which confirmed that ACE2 protects against MI; therefore, a relatively low serum ACE2 level shortly after CABG, may indicate a deficient protective mechanism against MI. This may explain why the risk of developing postoperative MI was significantly greater in the lowest serum ACE2 level quartile group following CABG. The troponin system has been observed to be extremely sensitive, however, not definitive (21). Therefore, future investigations are required to identify whether the serum ACE2 level may be a more specific marker than cardiac troponin levels for detecting postoperative MI.

Polymorphisms in the ACE2 gene are associated with the development of pathological myocardial hypertrophy and heart disease in humans (14). No significant associations were identified between the serum ACE2 level quartile groups and the ACE2 genotypes and haplotypes, in the present study, which were identified to be associated with MI in previous studies (14). Although the sample size may not be adequate for a definitive answer to address this issue (particularly in the female group), the findings reduced the possibility that the observed association between the serum ACE2 level and postoperative MI following CABG was a result of ACE2 polymorphisms.

The strength of the present study was due to the use of a relatively large sample size of patients who underwent CABG and the analysis results were adjusted for multiple relevant factors, including aortic clamp time, number of grafts and pre-CABG medication use. The findings indicated that post surgery, specific attention should be given to CABG patients exhibiting a relatively low serum ACE2 level (when based on an established population-specific normal referencing range) shortly after undergoing CABG, as there is a tendency to develop postoperative MI following CABG. In addition, the findings indicate that supplementing ACE2 levels may be a potential novel therapy for postoperative MI following CABG. The limitation of the present study was that it was conducted only with patients undergoing CABG with a CPB, and patients undergoing off-pump CABG was not included. This was because CABG with a CPB was the predominant type of CABG surgery with which we were able to recruit for an adequate sample size. The association between the serum ACE2 level and postoperative MI following off-pump CABG will be investigated in future studies, based on accumulating a sample of appropriate patients.

In conclusion, the present study indicated that the serum ACE2 level 1 h post CABG was independently associated with an increased risk of postoperative MI. Thus, observations of the serum ACE2 level may be a potential novel prognostic factor for postoperative MI, following CABG.

## Figures and Tables

**Table I:** General characteristics of study subjects.

	Serum ACE2 quartile categories, ng/ml (n=34)				
		
	≤1.06	1.07–1.42	1.43–1.85	≥1.86	P-value
Age (years)	63.5±4.5	64.2±3.8	63.2±3.4	63.7±4.2	0.29
Gender					0.83
Male, n (%)	27 (79.4)	29 (85.3)	26 (76.5)	27 (79.4)	
Female, n (%)	7 (20.6)	5 (14.7)	8 (23.5)	7 (20.6)	
Unstable angina	14 (41.2)	15 (44.1)	13 (38.2)	11 (32.4)	0.78
Hyperlipidemia, n (%)	31 (91.2)	31 (91.2)	32 (94.1)	30 (88.2)	0.87
Hypertension, n (%)	22 (64.7)	21 (61.8)	23 (67.6)	18 (52.9)	0.63
Diabetes mellitus, n (%)	6 (17.6)	5 (14.7)	7 (20.6)	5 (14.7)	0.90
Prior MI	9 (26.5)	10 (29.4)	8 (23.5)	8 (23.5)	0.94
BMI (kg/m^2^)	28.7±4.8	27.9±6.0	29.3±5.2	28.1±4.9	0.27
Current smoker	8 (23.5)	12 (35.3)	9 (26.5)	11 (32.4)	0.70

ACE, angiotensin-converting enzyme; MI, myocardial infarction; BMI, body mass index.

**Table II:** Disease and procedural characteristics of study subjects.

	Serum ACE2 quartile categories, ng/ml (n=34)				
		
	≤1.06	1.07–1.42	1.43–1.85	≥1.86	P-value
Disease pattern					
LM or LM+1 vessel, n (%)	3 (8.8)	4 (11.8)	3 (8.8)	2 (5.9)	0.87
LM+2 or 3 vessels, n (%)	5 (14.7)	4 (11.8)	4 (11.8)	3 (8.8)	0.90
Two vessel, n (%)	8 (23.5)	9 (26.5)	8 (23.5)	6 (17.6)	0.85
Three vessel, n (%)	18 (52.9)	17 (50.0)	19 (55.9)	23 (67.6)	0.48
Lesions (n)	3.1±0.2	3.0±0.3	3.0±0.3	3.2±0.4	0.83
Gensini score	37.5±4.6	37.3±5.1	37.4±4.8	37.0±3.9	0.92
Procedural characteristics					
On-pump, n (%)	34 (100.0)	34 (100.0)	34 (100.0)	34 (100.0)	1.00
CPB time (mins)	49.5±3.9	52.3±4.2	54.2±3.9	53.6±4.5	0.41
Aortic clamp time (mins)	32.5±1.9	31.9±2.0	33.2±2.1	32.9±2.7	0.53
Grafts (n)	2.5±0.2	2.4±0.2	2.5±0.1	2.5±0.2	0.95
LIMA use, n (%)	30 (88.2)	31 (91.2)	31 (91.2)	30 (88.2)	0.96
SVG use, n (%)	30 (88.2)	31 (91.2)	31 (91.2)	30 (88.2)	0.96
Free arterial graft use, n (%)	4 (11.8)	3 (8.8)	3 (8.8)	4 (11.8)	0.96
Pre-CABG medication					
Aspirin, n (%)	31 (91.2)	32 (94.1)	30 (88.2)	31 (91.2)	0.87
Clopidogrel, n (%)	4 (11.8)	6 (17.6)	5 (14.7)	5 (14.7)	0.93
Statin, n (%)	31 (91.2)	31 (91.2)	30 (88.2)	32 (94.1)	0.87
β-blocker, n (%)	26 (76.5)	28 (82.4)	27 (79.4)	25 (73.5)	0.84
ACE inhibitor/ARB, n (%)	21 (61.8)	19 (55.9)	19 (55.9)	20 (58.8)	0.95

Continuous variable values were expressed as the mean ± standard deviation. ACE, angiotensin-converting enzyme; ARB, angiotensin II receptor blocker; CABG, coronary artery bypass grafting; CPB, cardiopulmonary bypass; LIMA, left internal mammary graft; LM, left main; SVG, saphenous vein graft.

**Table III:** Adjusted HRs of postoperative MI by serum ACE2 levels.

	Serum ACE2 quartile categories, ng/ml (n=34)				P-value for trend (across categories)	Continuous log scale^a^	P-value for trend (continuous)

	≤1.06	1.07–1.42	1.43–1.85	≥1.86			
Postoperative MI, n (%)	9 (26.5)	5 (14.7)	4 (11.8)	2 (5.9)			
Model 1^b^ HR (95% CI)	5.76 (1.14–29.08)	2.76 (0.50–15.33)	2.13 (0.36–12.51)	1 (Reference)	<0.001	2.51 (2.06–2.92)	<0.001
Model 2^c^ HR (95% CI)	2.94 (1.85–4.16)	1.49 (0.92–2.58)	1.19 (0.57–2.11)	1 (Reference)	0.009	1.92 (1.53–2.27)	0.007

a HR per 1 - standard deviation decrease of log-transformed ACE2 levels;

b Model 1: Adjusted for age, gender and BMI;

c Model 2: Adjusted for age, gender, BMI, hypertension, prior MI, current smoking status, hyperlipidemia, diabetes mellitus, Gensini score, aortic clamp time, number of grafts and pre-coronary artery bypass grafting medications.

HR, hazard ratio; MI, myocardial infarction; ACE2, angiotensin-converting enzyme 2; BMI, body mass index.

**Table IV:** ACE2 levels with postoperative morbidities and in-hospital mortality.

		Serum ACE2 quartile categories (ng/ml)		
			
	Total (n=136)	≤1.06 (n=34)	>1.06 (n=102)	P-value
Postoperative MI	20 (14.7)	9 (26.5)	11 (10.8)	0.046^a^
Arrhythmia	37 (27.2)	14 (41.2)	23 (22.5)	0.045^a^
Low cardiac output	17 (12.5)	8 (23.5)	9 (8.8)	0.036^a^
Pulmonary complications	21 (15.4)	8 (23.5)	13 (12.7)	0.170
Neurologic complications	6 (4.4)	2 (5.9)	4 (3.9)	0.640
Excessive bleeding	3 (2.2)	1 (2.9)	2 (2.0)	1.000
In-hospital mortality	6 (4.4)	4 (11.8)	2 (2.0)	0.034^a^

a P<0.05. Data are expressed as n (%).

ACE2, angiotensin-converting enzyme 2; MI, myocardial infarction.

**Table V:** ACE2 polymorphisms and serum ACE2 levels.

A, Female (n=27)						

	Serum ACE2 quartile categories (ng/ml)					
			
ACE2 polymorphisms	≤1.06	1.07–1.42	1.43–1.85	≥1.86	Total	P-value
1075A/G (rs1978124)						0.87
AA	3	2	2	2	9	
Non-AA	4	3	6	5	18	
Total	7	5	8	7	27	
8790A/G (rs2285666)						0.85
AA	2	1	3	3	9	
Non-AA	5	4	5	4	18	
Total	7	5	8	7	27	
16854G/C (rs4646142)						0.73
GG	3	3	4	2	12	
Non-GG	4	2	4	5	15	
Total	7	5	8	7	27	

B, Male (n=109)						

	Serum ACE2 quartile categories (ng/ml)					
			
ACE2 polymorphisms	≤1.06	1.07–1.42	1.43–1.85	≥1.86	Total	P-value

1075A/G-8790A/G and 16854G/C haplotype						0.57
GGC	3	7	4	6	20	
Non-GGC	24	22	22	21	89	
Total	27	29	26	27	109	

ACE, angiotensin-converting enzyme.
